# Cas9-targeted-based long-read sequencing for genetic screening of *RPE65* locus

**DOI:** 10.3389/fgene.2024.1439153

**Published:** 2024-10-14

**Authors:** Cristina Rodilla, Gonzalo Núñez-Moreno, Yolanda Benitez, Raquel Romero, Lidia Fernández-Caballero, Pablo Mínguez, Marta Corton, Carmen Ayuso

**Affiliations:** ^1^ Department of Genetics and Genomics, Instituto de Investigación Sanitaria-Fundación Jiménez Díaz University Hospital, Universidad Autónoma de Madrid (IIS-FJD, UAM), Madrid, Spain; ^2^ Center for Biomedical Network Research on Rare Diseases (CIBERER), Instituto de Salud Carlos III, Madrid, Spain; ^3^ Bioinformatics Unit, Instituto de Investigación Sanitaria-Fundación Jiménez Díaz University Hospital, Universidad Autónoma de Madrid (IIS-FJD, UAM), Madrid, Spain

**Keywords:** *RPE65* gene, Leber congenital amaurosis, nanopore sequencing, CRISPR, retinitis pigmentosa

## Abstract

**Introduction:**

Long-read sequencing (LRS) enables accurate structural variant detection and variant phasing. When a molecular diagnosis is suspected, target enrichment can reduce the cost and duration of sequencing.

**Methods:**

LRS was conducted in five inherited retinal dystrophy (IRD) patients harboring a monoallelic variant in *RPE65* that remained uncharacterized after clinical exome sequencing (CES). CRISPR-Cas9 guide RNA probes were designed to target a 31 kb region, including the entire *RPE65* locus. The DNA was sequenced on a MinION platform. Short-read ×30 whole-genome sequencing (WGS) was performed for five patients to validate nanopore results.

**Results:**

The nanopore sequencing process yielded a median of 271 reads within the targeted region, with a mean depth of 109 and a median read size of 8 kb. All variants identified by CES have been detected using this approach, and no additional *RPE65* gene causative variants were found. Nanopore variant detection demonstrated performance akin to short-read WGS at similar coverage levels, although exhibiting increased false positive calls at lower coverage.

**Discussion:**

In this study, we explore the advantages of using a targeted approach together with long-read sequencing to identify variants associated with IRD. The results underscore the utility of targeted long reads for characterizing patients affected by rare diseases when first-tier diagnostic tests are non-conclusive.

## Introduction

Inherited retinal diseases (IRDs) are a group of clinically and genetically heterogeneous rare disorders characterized by a neurodegenerative process in retina cells, primarily of photoreceptors. IRDs cause progressive vision loss and may differ in the age of onset and severity of symptoms. The retinal pigment epithelium-specific 65 kDa (*RPE65*) gene is located in chromosome 1 and codifies for an enzyme implicated in the regeneration of the 11-cis-retinal chromophore during phototransduction in both rod and cone photoreceptors (Jacobson et al., 2007). Loss-of-function biallelic *RPE65* variants have been associated with two types of IRDs: Leber’s congenital amaurosis (LCA) and retinitis pigmentosa (RP).

In 2021, we reported *RPE65* biallelic variants to be the molecular cause of 2.6% of non-syndromic IRD in Spanish families (Lopez-Rodriguez et al., 2021). This prevalence is similar to the 2% reported in the Italian IRD cohort (Karali et al., 2022), the 2.3% in the Russian population (Stepanova et al., 2023), and higher than the 0.8% prevalence reported in a Chinese cohort (S. Li et al., 2020).

Individuals with biallelic *RPE65* variants are the first IRD cohort to undergo treatment with an approved gene-based therapy known as Voretigene Neparvovec (Patel et al., 2018). Molecular characterization of new *RPE65* patients would mean access to disease treatment options for these individuals.

Genome sequencing allows the identification of many genetic variants, including single-nucleotide variants (SNVs) in coding and non-coding regions, as well as structural variants (SVs) that are often missed by other molecular analyses. Long-read sequencing (LRS) can generate reads of 10–100 kb in size, which can encompass a complete SV in a single continuous read, allowing to understand its size, position and frequency in the population (Logsdon, Vollger and Eichler, 2020). The most widespread long-read technologies are single-molecule real-time (SMRT) sequencing by Pacific Biosciences (PacBio) (Rhoads and Au, 2015) and nanopore-based sequencing developed by Oxford Nanopore Technologies (ONT) (Jain et al., 2016). The latter represents a scalable and accessible option for smaller budgets.

Whole-genome approaches generate large quantities of data that require large storage capacity and lead to the possibility of secondary genetic findings. Targeted genomic approaches are a better alternative to identifying these variants when a clinically distinct condition is suspected (Khan et al., 2020).

Here, we describe the application of CRISPR-Cas9-mediated enrichment combined with ONT sequencing to the *RPE65* locus in undiagnosed IRD patients who are carriers of a heterozygous previously identified variant.

## Material and methods

### Patient selection and clinical diagnosis

Patients were recruited from the Fundación Jiménez Díaz (FJD) University Hospital (Madrid, Spain) inherited retinal dystrophy (IRD) cohort of 5,123 families from 1990 to February 2024 (March 2024 updated from Perea-Romero et al., 2021). Informed consent was obtained from all study subjects. This study adhered to the tenets of the Declaration of Helsinki and was approved by the FJD Research Ethics Committee (Approval No.: PIC172-20_FJD). For this study, we selected five patients who carried a heterozygous variant in *RPE65* identified after genetic testing by clinical exome sequencing (CES), as previously described (Perea-Romero et al., 2021). None of these patients carried an additional known pathogenic variant in 280 other genes associated with nonsyndromic and syndromic IRD or 18 candidate genes from the studied panels (Supplementary Table 1). Additionally, WGS data from these patients are being analyzed with an extended panel without relevant diagnostic progress.

Clinical diagnosis was based on ophthalmology findings and a specific questionnaire. The diagnosis of retinitis pigmentosa (RP) was assigned when the first symptoms were poor night vision and/or peripheral vision loss, as previously described in Perea-Romero et al., 2021. Leber’s congenital amaurosis (LCA) was diagnosed based on severe visual impairment in the first year of life, nystagmus, and non-recordable electroretinography responses.

### DNA extraction

High-molecular-weight genomic DNA (gDNA) was extracted from frozen blood cells using the EZ1 Advanced XL extraction system (QIAGEN) according to the manufacturer’s instructions. DNA concentration was assessed using Qubit double-stranded DNA assays (Thermo Fisher Scientific). The DNA quality was analyzed spectrophotometrically (NanoDrop ND-1000, Thermo Scientific), and integrity was ensured by gel electrophoresis.

### Short-read sequencing

The five selected patients were analyzed as a first-tier analysis using Clinical Exome Solution (CES, developed by Sophia Genetics, Boston, MA, United States). Libraries were prepared following the manufacturer's instructions and sequenced on a NextSeq500 platform (Illumina) (Martin-Merida et al., 2019).

The whole-genome sequencing (WGS) library was prepared from 1 µg of gDNA using NEB Next^®^ Ultra™ DNA Library Preparation Kit and sequenced paired-end (2 × 150 bp) in an Illumina Novaseq6000 sequencing platform at ×30 coverage. Raw sequencing reads were aligned to the GRCh37/hg19 assembly using the BWA Av 0.7.15 with default parameters.

### Guide RNA design

The Alt-R™ CRISPR-Cas9 System (Integrated DNA Technologies, IDT) was selected to design and assemble the CRISPR RNAs (crRNA) and the trans-activating crRNAs (tracrRNA) into functional guide RNAs (gRNAs). crRNA sequences were identified using the CHOPCHOP tool (available at chopchop.cbu.uib.no; Labun et al., 2019).

**FIGURE 1:**
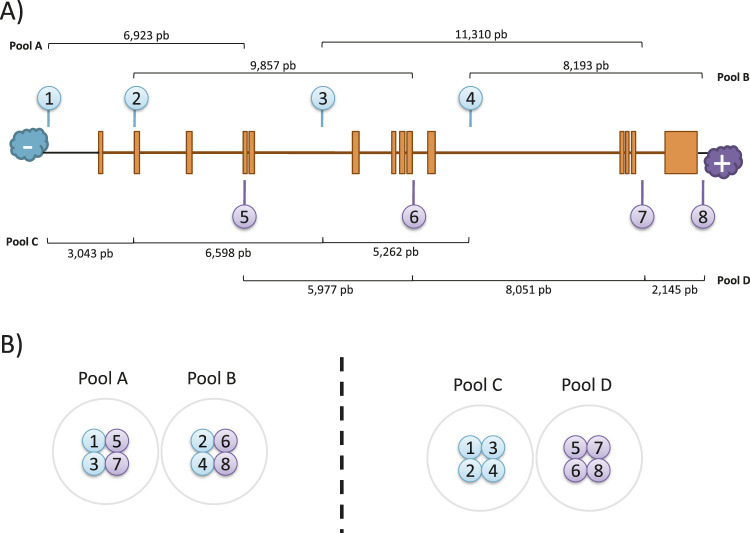
CRISPR-Cas9 enrichment design. **(A)** Schematic representation of gRNAs location in the target region of *RPE65*. The distances between expected cuts were calculated for each pool. **(B)** Arrangement of gRNAs for library preparation.

The crRNA sequences were designed following the Oxford Nanopore Technologies (ONT) guidelines following a “tiling” approach as recommended for regions of interest larger than 20 kb. Thus, pairs of probes were designed for targeting in 10 kb overlapping blocks to achieve uniform coverage along a 31 kb region (chr1:68,423,822-68,454,954; GRCh38/hg38) that included the full-size *RPE65* locus (chr1:68,428,822-68,449,954; GRCh38/hg38) and flanking 5 kb expanded regions on each end. A total of six gRNAs could be designed, and the theoretically best-performing gRNAs were selected based on the following criteria: i) a GC content between 30% and 80%; ii) a self-complementarity equal to 0; iii) off-target mismatches lower than 10; iv) a theoretic cut efficiency ≥0.2. In addition, the final selection of probes was made based on the complementary strand where the cut will be performed, the availability of a guide pair within a 10 kb distance in the other strand, the formation of overlapping clusters with other probes, and the best overall parameters predicted by CHOPCHOP. Finally, four gRNA pairs were selected, targeting both positive and negative strands (Table 1; Figure 1A), including two cutting upstream and downstream *RPE65* loci, and six within intragenic sequences, mostly in intronic regions.

**TABLE 1:** Location and characteristics of the selected gRNAs for CRISPR-Cas9-mediated targeted capture of the *RPE65* locus. MM: off-target mismatches.

Name	Strand	Sequence	*RPE65* location	%GC	Self-complementarity	MM0	MM1	MM2	MM3	Efficiency
1	−	GTA​TCA​ATG​TTC​TTC​CTG​GGT​GG	Flanq 5′	45	0	0	0	0	7	0.69
2		CGG​ACT​TTG​AGC​ATC​AAC​ATG​GG	Intron 1	45	0	0	0	0	5	0.38
3		CGG​CTT​ATT​GGT​CTA​ATG​CAG​GG	Intron 5	45	0	0	0	0	4	0.42
4		GGT​ATC​TAA​TTA​CAT​GTG​AGG​GG	Intron 10	35	0	0	0	0	4	0.27
5	+	CCC​GTA​CGT​AAG​CAT​CAG​TGC​GG	Exon 4	55	0	0	0	0	1	0.6
6		GTA​AAA​ACC​CCG​TAA​TTT​CCA​GG	Intron 9	40	0	0	0	0	2	0.24
7		TGC​TCC​ATC​GTG​ACA​CCA​AAT​GG	Intron 13	50	0	0	0	0	7	0.68
8		AAT​GGA​TAC​ATC​AGG​TAC​CCT​GG	Flanq 3′	45	0	0	0	0	8	0.21

### CRISPR-Cas9 enrichment

Library preparation was carried out following the ONT Cas9 Sequencing Kit (SQK-CS9109) protocol. The tiling approach requires preparing separate libraries with two sets of probes until the final steps. Probes were mixed in equimolar quantities to prepare the pools. From a pool, 1 µL was aliquoted and mixed with tracrRNA. The mix was incubated at 95°C for 5 min. Then, ribonucleoprotein complexes (RNPs) were formed by adding Alt-R™ S. p. Cas9 Nuclease (IDT) and incubating for 30 min.

Pool mixes were prepared in two ways to perform the *RPE65* enrichment library. The first way was the “bricklayer” approach (Library A-B, Figure 1B), where overlapping pairs were separated between pools, and the second way was the “highway” approach (Library C-D) where probes were separated according to the direction of the cut.

On account of using the tiling approach, >1,000 ng of gDNA was used for each experiment (>500 ng for each pool of RNPs). The sample was divided into two equimolar reactions for each RNP pool; a minimum of 1.5 µg of DNA for each pool was ensured to be prepared. Following purification, pools were combined into a single library for sequencing. DNA dephosphorylation, cleaving, dA-tailing, adapter ligation, and purification of target DNA were performed according to the protocol.

### Nanopore sequencing

Sequencing was carried out on a MinION flow cell (R9.4.1 FLO-MIN106). The MinION sequencer was run for 24 h using MinKNOW software (version 21.06.10). Basecalling was performed using Guppy (version 5.0.13) with the super-high accuracy model. Read filtering was established at Qscore 9, and filtering by read length was disabled.

### Sequencing analysis

Fastq files from short-read sequencing were aligned to the GRCh38/hg38 genome assembly using BWA software (H. Li and Durbin, 2009). SNVs and indels (small insertions and deletions) were called using the HaplotypeCaller function in the GATK genomic analysis toolkit (McKenna et al., 2010). Copy-number variants (CNVs) were called using the commercial SOPHiA DDM platform (Sophia Genetics) for CES, and SVs were called using Manta (Chen et al., 2016) for WGS analysis. Variant annotation was performed using an *in-house* pipeline, *NextVariantFJD*, available at https://github.com/TBLabFJD/NextVariantFJD (Romero et al., 2022) that includes the variant effect predictor (VEP) (McLaren et al., 2016) for SNVs and indels and AnnotSV (Geoffroy et al., 2018) for SVs, plus additional custom information.

Fastq files from nanopore sequencing were aligned to the GRCh38/hg38 genome assembly using minimap2 (Li, 2018). SNVs and indels were called using PEPPER (PEPPER-Margin-DeepVariant, Shafin et al., 2021) and annotated using the NextVariantFJD pipeline. SVs were called and annotated following the wf-human-variation workflow from EPI2ME Labs, available at https://github.com/epi2me-labs/wf-human-variation, which performs Sniffles2 (Smolka et al., 2024) as the SV caller and SnpEff (Cingolani et al., 2012) for the annotation.

To compare quality statistics from different sequencing methods, we generated two bed files containing 1) the full gene for WGS and nanopore sequencing, and 2) the coding regions of the canonical transcript (NM_000329.3) as described in NCBI for CES sequencing. Mosdepth (Pedersen and Quinlan, 2018) was used to obtain depth and coverage statistics using bam files and the corresponding bed file. The number of reads and N50 reads were calculated using NanoStat (De Coster and Rademakers, 2023) from bam files restricted to the regions in the custom bed files. The variant files, vcfs, were compared using tabix to extract the region of interest and merged using a custom Python script.

Sequencing data were first tested for previously identified variants as a control step of the reading quality achieved in the experiments. To select possible causative variants, the following criteria were applied following the American College of Medical Genetics and Genomics (ACMG) guidelines (Richards et al., 2015). Disease associations reported in the Human Gene Mutation Database (Professional 2022.1), the Leiden Open Variation Database (https://databases.lovd.nl/shared/genes/RPE65), and ClinVar were explored, along with a review of relevant literature. Allele frequency <0.001 in general population databases such as GnomAD v4.1.0 (Genome Aggregation Database; http://gnomad.broadinstitute.org/), Kaviar (Known VARiants) and CSVS (Collaborative Spanish Variant Server). To assess variant impact on protein function, *in silico* analysis was performed using 20 pathogenicity assessment tools (including CADD, SIFT, Provean, etc.) and splicing predictors (SpliceAI, Ada score, Rf score, MaxEntScan).

## Results

### Selective enrichment of *RPE65* locus sequencing

Our objective was to create a simple and effective method that facilitates fast and accurate characterization of *RPE65*-related IRD patients. The selected study cohort encompassed previously uncharacterized heterozygous patients with associated *RPE65* conditions, LCA, or RP (Table 2).

**TABLE 2:** Summary of patient information and library preparation for each experiment.

Patient		1	2	3	4	5
Clinical diagnosis		LCA	RP	RP	RP	RP
Variant change	Nucleotide	c.743A > G	c.1543C > T	c.292_311del	c.718G > T	c.1034A > G
	Protein	p.Asn248Ser	p.Arg515Trp	p.Ile98His fs*26	p.Val240Phe	p.Asn345Ser
	Pathogenicity classification	3	5	5	5	3
	ACMG criteria	PM2, PP2, PP3	PS3, PM2, PM3, PP2, PP3, PP5	PVS1, PM2, PM3, PP5	PM1, PM2, PM3, PP1, PP2, PP5	PM1, PM2, PP2, PP3
	Reference	NA	Kondo, 2004	Thompson, 2000	Glen, 2019	NA
Sample DNA	Quantity input (μg)	9.96	8.08	7.50	9.36	3.20^a^
	Abs 260/280	1.75	1.75	2.08	1.82	1.63
	Abs 260/230	1.26	1.02	0.89	0.89	0.75
	Integrity	Ok	Ok	Ok	Ok	Slightly degraded^a^
Nanopore sequencing	Initial pore availability	1,118	1,587	1,570	1,619	1,336
	Library	AB	AB	CD	CD	CD

Variants were annotated referring to the NM_000329.3 transcript. DNA integrity was evaluated as Ok if no signs of degradation were present in the gel electrophoresis and as “slightly degraded” if signs of degradation were observed. NA, Not applicable.

^a^ Quantity of DNA was limited for Patient 5, and it was slightly degraded.

To comprehensively sequence the entire genomic loci of *RPE65* using long reads, we selected eight specific crRNAs to perform nanopore sequencing. The crRNAs were designed to be located less than 10 kb away from each other, with pairs cutting on opposite strands at a mean distance of 9 kb, to ensure complete coverage of the region (Figure 1A).

**FIGURE 2:**
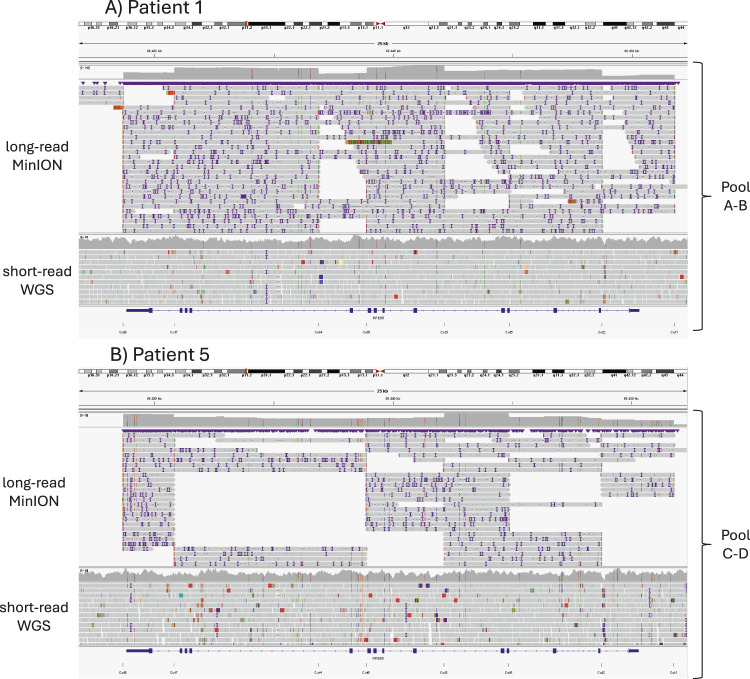
Alignment of long-read MinION sequencing reads using CRISPR-Cas9 enrichment (top) and short-read Illumina WGS reads (bottom); the expected CRISPR-Cas9 cuts are shown below. **(A)** Results from Patient 1, for whom the library was prepared using pools A and B. **(B)** Results from Patient 5 using pools C and D.

Focusing on the library protocols, the sequencing depth achieved using the AB library was 1.78 times greater than that obtained with the CD library (Figure 2; Supplementary Table 2). The mean read size (N50) was also greater for the AB library, a result attributed to the experimental design.

### Variant detection

Patients were previously studied with CES and eight coding variants were identified within the patient samples. Using nanopore enrichment sequencing, we successfully identified all genetic variations that had been identified using CES (Supplementary Table 3).

No structural variant in the *RPE65* locus was detected in the studied patients. However, a total of 71 SNVs and indels were detected in the studied patients using nanopore: 12.68% (9/71) were coding variants, 83.09% (59/71) were deep intronic variants, and 4.23% (3/71) were located in the 3′UTR region. Seven variants presented an allele frequency of less than 0.1% and of them, only the five previously reported variants were predicted to cause a deleterious effect with supporting *in silico* predictions. The remaining variants (88%, 66/71) have not been associated with pathogenicity, and more than half (42/66) were considered polymorphisms due to having an allele frequency higher than 1% in GnomAD.

Short-read WGS detected 69 variants, and 98.55% (68/69) were also detected using CRISPR-Cas9 nanopore sequencing. However, three variants were exclusively detected with nanopore in patients with WGS studies. These variants were reported to have low quality (ranging from 4 to 10), and for this reason, they were considered false positives. False positive detection was increased in experiments with lower coverage (Patient 5). There was also one indel variant exclusively detected in WGS for Patient 5. This variant implied a one nucleotide duplication and was detected in four of 19 reads for the long-read experiment, although the variant caller did not call it.

### Comparison of targeted long-read data with short-read data

To further evaluate the different methodologies used, we have compared the sequencing data obtained with each approach.

Nanopore sequencing obtained 271 mean reads, while short-read technologies produced more than 7 times as many for CES and 17 times as many for WGS (Table 3). Short reads had a standard size of 150 bp, while nanopore long reads reached more than 8,000 bp.

**TABLE 3:** Sequencing statistics of studied patients. N detected SNVs + indels: number of detected SNVs and indels.

		Nanopore		CES		WGS	
	Patient	Values	Mean	Values	Mean	Values	Mean
Reads	1	301	271	2,131	2,032	4,467	4,574
	2	328		2,166		4,354	
	3	456		1,573		4,573	
	4	185		1,993		5,156	
	5	84		2,297		4,322	
PASS read N50 (bp)	1	9,659	8,349	151	151	151	151
	2	9,603		151		151	
	3	8,033		151		151	
	4	6,523		151		151	
	5	7,926		151		151	
Min depth	1	18	9.60	38	33.8	10	10
	2	5		37		6	
	3	19		26		13	
	4	5		33		15	
	5	1		35		6	
Mean depth	1	96.38	73.68	84.26	80.1	28.79	29.27
	2	102.48		85.99		28.07	
	3	115.13		63.3		29.47	
	4	34.51		73.35		32.79	
	5	17.9		93.58		27.21	
Max depth	1	146	109.2	136	121.4	47	48.4
	2	143		130		46	
	3	165		91		51	
	4	66		113		52	
	5	26		137		46	
N Detected SNVs + Indels	1	14	29	1	1.8	14	28.8
	2	17		2		16	
	3	40		2		41	
	4	35		2		35	
	5	39		2		38	

Minimum coverage in the region was around 30 for CES and dropped to 10 and 9.6 reads for WGS and nanopore, respectively. Mean coverage obtained in the *RPE65* locus varied between experiments, ranging from 18 to ×115 between nanopore experiments. CES studies had a similar mean coverage to nanopore, and WGS depth was close to 30. Nanopore reached a maximum of ×109, with one experiment having up to ×165 (Table 3).

**FIGURE 3:**
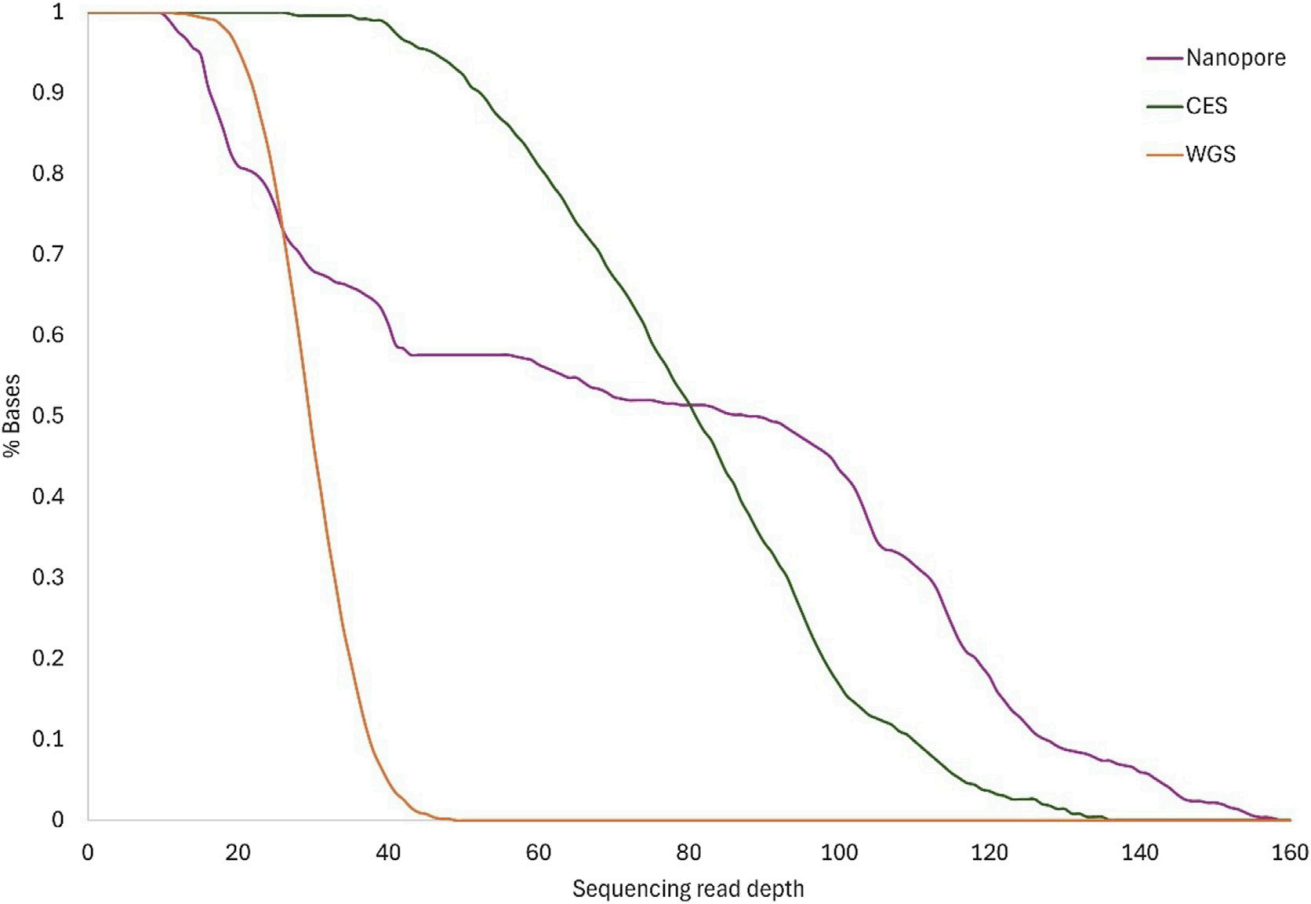
Sequence coverage. The lines show the cumulative read depth as a percentage of total bases for each method: Nanopore, CES, and WGS. Lines show the mean coverage for the same method between the different samples.

On average, 81% of the RPE65 locus had coverage of at least ×20 using the nanopore method. More than ×60 mean nanopore coverage was reached in at least 56% of the RPE65 region, which was more than obtained with WGS (Figure 3). Although the minimum depth obtained with nanopore sequencing was similar to WGS, the resulting depth reaches higher values using a targeted approach (Table 3).

## Discussion

In this study, we demonstrated that the implementation of Cas9-mediated long-read sequencing can explore the *RPE65* locus in depth for IRD patients.

Biallelic variants in the *RPE65* gene are the molecular cause of 2.6% of IRD families in Spain (Lopez-Rodriguez et al., 2021). To fully characterize any potential *RPE65* patient, we have applied this technology to determine if a second allele was present in the remaining five monoallelic *RPE65*-uncharacterized IRD families in our cohort (0.26%, 5/5123 uncharacterized IRD families). The importance of investigating monoallelic patients in the context of a recessive disorder lies in the potential of identifying a second hit in the same gene, potentially leading to a definitive molecular diagnosis for the individual. This holds particular significance for the *RPE65* gene because therapeutic intervention is already available (Fischer et al., 2024). By performing this analysis, we can rule out biallelic *RPE65* single-nucleotide variants as the molecular cause of IRD in the studied cohort, thereby refining our understanding of the genetic mechanisms contributing to the disorder.

Previously detected variants were identified in all studied patients with this new method, even at a ×17 mean read depth. Although the bricklayer strategy (library AB) obtained longer reads and more sequencing depth throughout the region, differences could not be attributed exclusively to library preparation because the two protocols were not compared in the same samples, and the number of samples was small. In addition, sequencing yield might have been affected for Patient 5 due to low integrity DNA and limited quantity. Almost all variants (98.55%) detected by second generation sequencing were also identified with long-read nanopore sequencing.

Nanopore sequencing using R9 chemistry has higher error rates (Q20 accuracy) than short-read NGS sequencing (Wang et al., 2021) or similar third-generation sequencing technologies such as PacBio (Lang et al., 2020). In our hands, only three variants were exclusively detected in nanopore sequences. However, the most recent R10 nanopore chemistry achieves an F1-score, accuracy, and false-discovery rate closer to Illumina sequencing (Kolmogorov et al., 2023; Ni et al., 2023). Although the latest ONT basecaller, Dorado, outperforms the Guppy basecaller (Kuśmirek, 2023), it could not be directly tested in our study because raw data were unavailable. Additionally, ONT reads are reported to reach higher contiguity assemblies (Lang et al., 2020). A standard method such as Sanger sequencing is recommended for variant confirmation when no previous technology is available to validate the variant results.

When assessing the cost-effectiveness of a new technology, it is essential to look beyond the price of reagents (library kits and flowcell) and consider the broader financial implications. Enrichment sequencing using nanopore technology costs approximately 900€ per sample. This is more expensive than NGS techniques such as CES and WGS, which cost between 200€ and 600€. However, these prices do not consider direct labor costs and sequencing analysis, which greatly increase the overall price because NGS libraries take at least 3 days to prepare and comprehend the analysis of thousands of variants. Nanopore enrichment sequencing only takes 6 h to prepare and a day to complete sequencing. Furthermore, variants can be analyzed from the start of the sequencing, and because it is an enrichment approach, only an average of 28 variants need to be interpreted. For this reason, the time saved through nanopore technology translates directly into cost savings and increased productivity.

CRISPR-Cas9 enrichment using nanopore sequencing limits the analysis scope to the selected targeted region. Any variants or structural rearrangement breakpoints localized further from the end of the region would not be captured even if they affect the function of the gene.

Alternative targeted enrichment techniques are PCR-dependent (McClinton et al., 2023), which has length limitations, may introduce artifacts, or produce allele-biased amplification. Another amplification-free enrichment technique is ReadFish adaptive sampling (Payne et al., 2021), which is recommended for larger targets of >3 Mb. Moreover, adaptive sampling requires library reload on a MinION flow cell (Nakamichi et al., 2023) or a run in a more powerful and expensive device using a PromethION flow cell (Deserranno et al., 2023), limiting implementation due to sample availability and cost constraints. Lastly, low-coverage long-read WGS might be effective for characterizing structural variants (Lavrichenko, Johansson, and Jonassen, 2021), but the detection of single-nucleotide variants could be affected.

In conclusion, nanopore sequencing could be considered a robust and feasible system for finding or implementing a second allele strategy in monoallelic recessive cases, with good sensitivity due to the fact that it was able to detect previously identified variants. Additionally, in the context of patient care, using such advanced technologies can significantly improve patients’ clinical management by enabling faster and more accurate molecular diagnosis. This leads to personalized treatment options and genetic counselling, ultimately improving overall healthcare quality and resulting in better health outcomes.

## Data Availability

The original contributions presented in the study are publicly available. This data can be found here: European Genome-phenome Archive (EGA) repository, accession numbers: EGAS50000000596 and EGAD50000000847.
