# Supplementary material for: Cas9-targeted-based long-read sequencing for genetic screening of RPE65 locus
Source: Front Genet. 2024 Oct 14;15:1439153. doi: 10.3389/fgene.2024.1439153 (PMC11513366; doi:10.3389/fgene.2024.1439153)
Supplement: Supplementary file 1 [file Table1.docx]

**Supplementary Table 1. Summary of the list of genes included for the analysis.**

| **IRD non-syndromic and syndromic panel** |
| --- |
| *ABCA4, ABCC6, ABHD12, ACBD5, ACO2, ACTB, ACTG1, ADAM9, ADAMTS18, ADGRV1, AFG3L2, AHI1, AIPL1, AIRE, ALDH3A2, ALMS1, AMACR, ANTXR1, ARL13B, ARL3, ARL6, ATF6, ATOH7, B9D1, B9D2, BBS1, BBS10, BBS12, BBS2, BBS4, BBS5, BBS7, BBS9, BCOR, BEST1, BMP4, C8orf37, CA4, CABP4, CACNA1F, CACNA2D4, CC2D2A, CDH23, CDH3, CDHR1, CEP164, CEP290, CEP41, CERKL, CHM, CISD2, CLN3, CLN5, CLN6, CLN8, CLRN1, CNGA1, CNGA3, CNGB1, CNGB3, CNNM4, COL11A1, COL11A2, COL18A1, COL2A1, COL4A1, COL9A1, COL9A2, COL9A3, CRB1, CRX, CRYAB, CTC1, CTNNB1, CTSD, CYP27A1, CYP4V2, DHDDS, DNAJC5, DYNC2H1, EDNRB, EFEMP1, ELOVL4, ERCC1, ERCC2, ERCC6, ERCC8, EYA1, EYS, FAM161A, FLVCR1, FRAS1, FREM2, FSCN2, FZD4, GALE, GLA, GLI2, GLIS2, GNAT1, GNAT2, GNB3, GNPTG, GPR143, GPR179, GRIP1, GRK1, GRM6, GRN, GUCA1A, GUCA1B, GUCY2D, HARS, HCCS, HESX1, HGSNAT, HK1, HMX1, IDH3B, IDS, IFT140, IFT172, IFT80, IMPDH1, IMPG2, INPP5E, IQCB1, KCNJ13, KCNV2, KIF11, KIF1A, KLHL7, LAMA1, LCA5, LMX1B, LRAT, LRIT3, LRP2, LRP5, LZTFL1, MAK, MERTK, MFRP, MFSD8, MITF, MKKS, MKS1, MMACHC, MTTP, MYH9, MYO7A, NAA10, NDP, NEK8, NEUROD1, NHS, NMNAT1, NPHP1, NPHP3, NPHP4, NR2E3, NR2F1, NRL, NYX, OAT, OCRL, OFD1, OTX2, P3H2, PACS1, PANK2, PAX2, PAX3, PAX6, PCARE, PCDH15, PDE6A, PDE6B, PDE6C, PDE6G, PDZD7, PEX1, PEX2, PEX6, PEX7, PHYH, PLA2G5, PNPLA6, POMGNT1, PORCN, PPT1, PRCD, PROM1, PRPF3, PRPF31, PRPF6, PRPF8, PRPH2, PRPS1, RAB18, RAB3GAP1, RAB3GAP2, RAX2, RBP3, RBP4, RCBTB1, RD3, RDH12, RDH5, RGR, RGS9, RHO, RIMS2, RLBP1, ROM1, RP1, RP1L1, RP2, RP9, RPE65, RPGR, RPGRIP1, RPGRIP1L, RS1, SAG, SALL4, SDCCAG8, SEMA3E, SHH, SIL1, SLC24A1, SLC33A1, SLC41A1, SLC52A2, SLC6A6, SLC9A6, SMOC1, SNAI2, SNRNP200, SOX10, SOX2, SOX3, SPATA7, SPG7, SUFU, TCTN1, TCTN2, TFAP2A, THRB, TIMM8A, TIMP3, TINF2, TMEM138, TMEM216, TMEM231, TMEM237, TOPORS, TPP1, TREX1, TRPM1, TSPAN12, TTC8, TTPA, TUB, TUBGCP6, TULP1, UNC119, USH1C, USH1G, USH2A, VCAN, VPS13B, WDPCP, WDR19, WFS1, WHRN, XPNPEP3, ZFYVE26, ZNF423, ZNF513.* |
| **Candidate gene panel** |
| *ADIPOR1, AHR, ATP1A3, COQ2, COQ5, ESPN, FBN1, FRMD7, GDF6, JAG1, MVK, NBAS, OPN1SW, PDE6H, PDSS1, PGK1, SEMA4A, VSX2.* |
