# Supplementary material for: Cas9-targeted-based long-read sequencing for genetic screening of RPE65 locus
Source: Front Genet. 2024 Oct 14;15:1439153. doi: 10.3389/fgene.2024.1439153 (PMC11513366; doi:10.3389/fgene.2024.1439153)
Supplement: Supplementary file 2 [file Table2.docx]

**Supplementary Table 2. Differences within nanopore sequencing experiments.** Mean coverage and N50 reads values obtained for each patient are classified according to library used in the experiment. No statistical test was conducted due to small number of experiments performed.

|  | **Library** | | | |
| --- | --- | --- | --- | --- |
|  | **AB** | | **CD** | |
|  | **Values** | **Mean** | **Values** | **Mean** |
| Mean depth | 96.38 | 99.43 | 115.13 | 55.85 |
|  | 102.48 |  | 34.51 |  |
|  |  |  | 17.9 |  |
| Read N50 (bp) | 9659 | 9631 | 8033 | 7494 |
|  | 9603 |  | 6523 |  |
|  |  |  | 7926 |  |
