# Supplementary material for: Cas9-targeted-based long-read sequencing for genetic screening of RPE65 locus
Source: Front Genet. 2024 Oct 14;15:1439153. doi: 10.3389/fgene.2024.1439153 (PMC11513366; doi:10.3389/fgene.2024.1439153)
Supplement: Supplementary file 3 [file Table3.docx]

**Supplementary Table 3. Detected variants in *RPE65* locus.** Variant detection within the three sequencing strategies, MinION, CES and WGS. Each patient carrying the specific variant is named as P1-P5 for Patient 1-5. GnomAD MAF, total frequency of the alternate allele in GnomAD. Max Splice AI, maximum score value of Splice AI regarding the variant. Previously reported variants in studied patients are highlighted in grey.

| Variant | Nucleotide change | GnomAD MAF | CADD Phred | Max SpliceAI | MinION | CES | WGS |
| --- | --- | --- | --- | --- | --- | --- | --- |
| 1-68449442-C-T | c.11+453G>A | 0.01987 | 2.38 | 0 | P5 |  | P5 |
| 1-68449261-A-G | c.12-555T>C | 0.1128 | 4.24 | 0 | P5 |  | P5 |
| 1-68449034-C-G | c.12-328G>C | 0.005367 | 0.503 | 0 | P3 |  | P3 |
| 1-68448987-A-G | c.12-281T>C | 0.05888 | 0.254 | 0 | P3 |  | P3 |
| 1-68448323-C-T | c.94+301G>A | 0.1102 | 0.58 | 0 | P3 |  | P3 |
| 1-68447894-C-A | c.94+730G>T | 0.413 | 1 | 0 | P3;P4;P5 |  | P3;P4;P5 |
| 1-68447776-T-G | c.94+848A>C | 0.4175 | 1.17 | 0 | P3;P4;P5 |  | P3;P4;P5 |
| 1-68447254-T-G | c.95-394A>C | 0.1882 | 0.542 | 0 | P3 |  | P3 |
| 1-68447099-A-C | c.95-239T>G | 0.003448 | 1.08 | 0 | P5 |  | P5 |
| 1-68447072-T-C | c.95-212A>G | 0.4174 | 0.537 | 0.01 | P3;P4;P5 |  | P3;P4;P5 |
| 1-68446988-C-T | c.95-128G>A | 0.001822 | 4.78 | 0 | P5 |  | P5 |
| 1-68446330-T-TGCTA | c.245+376_245+379dup | 0.14 | 0.422 | 0 | P3 |  | P3 |
| 1-68445316-C-A | c.246-433G>T | 0.39 | 0.43 | 0 | P1;P2;P3;P4;P5 |  | P1;P2;P3;P4;P5 |
| 1-68444929-C-T | c.246-46G>A | 0.01553 | 0.102 | 0 | P2 |  | P2 |
| 1-68444817-GCCAAATTCTGTTATGACGAT-G | c.292_311del | 1.98E-05 | 32 | 0.43 | P3 | P3 | P3 |
| 1-68443820-G-A | c.495+711C>T | 0.05924 | 1.11 | 0 | P1;P2 |  | P1;P2 |
| 1-68443685-CT-C | c.495+845del | 0.001768 | 2.18 | 0 | P5 |  | P5 |
| 1-68443068-A-G | c.495+1463T>C | 0.001543 | 2.02 | 0 | P5 |  | P5 |
| 1-68442818-T-C | c.495+1713A>G | 0.4289 | 0.855 | 0 | P3;P4;P5 |  | P3;P4;P5 |
| 1-68441844-A-C | c.496-844T>G | 0.5427 | 0.168 | 0 | P1;P2;P3;P4;P5 |  | P1;P2;P3;P4;P5 |
| 1-68441358-G-T | c.496-358C>A | 0.001768 | 0.665 | 0 | P5 |  | P5 |
| 1-68440586-C-CAT | c.643+265_643+266dup | 0.003017 | 0.607 | 0 | P5 |  | P5 |
| 1-68439863-C-A | c.644-221G>T | 0.001787 | 1.06 | 0 | P5 |  | P5 |
| 1-68439684-AT-A | c.644-43del | 0.01123 | 0.48 | 0.02 | P5 |  |  |
| 1-68439675-G-C | c.644-33C>G | 0.4499 | 0.801 | 0 | P1;P2;P3;P4;P5 |  | P1;P2;P3;P4;P5 |
| 1-68439568-C-A | c.718G>T | 3.47E-05 | 22.7 | 0.1 | P4 | P4 | P4 |
| 1-68439306-T-C | c.743A>G | 8.057E-06 | 25.5 | 0.14 | P1 | P1 | P1 |
| 1-68439059-T-G | c.881A>C | 0.00131 | 26.6 | 0 | P2 | P2 | P2 |
| 1-68438962-C-A | c.978G>T | 0.0004814 | 6.71 | 0.02 | P5 | P5 | P5 |
| 1-68438830-A-G | c.998+112T>C | 0.1354 | 2.94 | 0.02 | P3;P4 |  | P3;P4 |
| 1-68438735-A-G | c.998+207T>C | 0.9683 | 1.73 | 0 | P1;P2;P3;P4;P5 |  | P1;P2;P3;P4;P5 |
| 1-68438672-G-T | c.998+270C>A | 0.4288 | 0.026 | 0 | P3;P4;P5 |  | P3;P4;P5 |
| 1-68438645-GAGA-G | c.998+294_998+296del | 0.001536 | 0.464 | 0 | P5 |  | P5 |
| 1-68438583-T-C | c.999-267A>G | 0.5409 | 0.59 | 0 | P1;P2;P3;P4;P5 |  | P1;P2;P3;P4;P5 |
| 1-68438505-T-A | c.999-189A>T | 0.001767 | 4.17 | 0 | P5 |  | P5 |
| 1-68438281-T-C | c.1034A>G | 3.098E-06 | 26.6 | 0.14 | P5 | P5 | P5 |
| 1-68438259-C-T | c.1056G>A | 0.1343 | 6.06 | 0.09 | P3;P4 | P3;P4 | P3;P4 |
| 1-68438244-G-A | c.1071C>T | . | 9.183 | 0.26 | P5 |  |  |
| 1-68437940-G-A | c.1128+247C>T | 0.00001314 | 1.73 | 0 | P5 |  |  |
| 1-68436720-G-A | c.1128+1467C>T | 0.0843 | 0.347 | 0 | P3;P4 |  | P3;P4 |
| 1-68436715-G-A | c.1128+1472C>T | 0.2968 | 0.773 | 0 | P3;P4 |  | P3;P4 |
| 1-68436702-G-A | c.1128+1485C>T | 0.1612 | 0.851 | 0 | P3;P4 |  | P3;P4 |
| 1-68436554-T-C | c.1128+1633A>G | 0.5419 | 0.822 | 0 | P1;P2;P3;P4;P5 |  | P1;P2;P3;P4;P5 |
| 1-68436444-CTATTTATT-C.CTATTTATTTATT | c.1128+1735_1128+1742del. c.1128+1739_1128+1742dup | 0.2289, 0.05036 | 1.19, 1.12 | 0,0 | P4 |  | P4 |
| 1-68436444-CTATTTATTTATT-C | c.1128+1731_1128+1742del | 0.005645 | 1.2 | 0 | P5 |  | P5 |
| 1-68436444-CTATTTATT-C | c.1128+1735_1128+1742del | 0.2289 | 1.19 | 0 | P3 |  | P3 |
| 1-68436309-T-C | c.1128+1878A>G | 0.1614 | 1.86 | 0 | P3;P4 |  | P3;P4 |
| 1-68436228-T-C | c.1128+1959A>G | 0.4284 | 2.81 | 0 | P3;P4;P5 |  | P3;P4;P5 |
| 1-68436172-G-A | c.1128+2015C>T | 0.543 | 0.737 | 0 | P1;P2;P3;P4;P5 |  | P1;P2;P3;P4;P5 |
| 1-68436052-C-A | c.1128+2135G>T | 0.5428 | 0.164 | 0 | P1;P2;P3;P4;P5 |  | P1;P2;P3;P4;P5 |
| 1-68435958-A-G | c.1128+2229T>C | 0.001805 | 3.41 | 0 | P5 |  | P5 |
| 1-68435749-T-G | c.1128+2438A>C | 0.1519 | 2.89 | 0 | P3;P4 |  | P3;P4 |
| 1-68435174-GCTCTTGTTTCTTTTTCTGGCTT-G | c.1128+2991_1128+3012del | 0.5405 | 6.33 | 0 | P1;P2;P3;P4;P5 |  | P1;P2;P3;P4;P5 |
| 1-68434719-C-CTGTT | c.1129-3138_1129-3135dup | 0.05744 | 1.64 | 0 | P1;P2 |  | P1;P2 |
| 1-68434719-CTGTT-C | c.1129-3138_1129-3135del | 0.0557 | 1.98 | 0 | P4 |  | P4 |
| 1-68434556-T-C | c.1129-2971A>G | 0.4449 | 3.5 | 0 | P3;P4;P5 |  | P3;P4;P5 |
| 1-68434115-T-C | c.1129-2530A>G | 0.2492 | 3.74 | 0 | P2 |  |  |
| 1-68434115-TACACAC-T | c.1129-2536_1129-2531del | 0.002301 | 0.73 | 0 | P5 |  | P5 |
| 1-68434113-TATAC-T | c.1129-2532_1129-2529del | 0.136 | 0.645 | 0 | P3;P4 |  | P3;P4 |
| 1-68434091-GAT-G | c.1129-2508_1129-2507del | 0.2044 | 0.688 | 0 | P2;P5 |  | P3;P2;P5 |
| 1-68434091-GATAT-G | c.1129-2510_1129-2507del | 0.01097 | 0.678 | 0 | P1 |  | P1 |
| 1-68434079-G-A | c.1129-2494C>T | 0.0721 | 0.078 | 0 | P1;P2 |  | P1;P2 |
| 1-68433977-G-A | c.1129-2392C>T | 0.2617 | 1.71 | 0 | P3;P4;P5 |  | P3;P4;P5 |
| 1-68433703-A-G | c.1129-2118T>C | 0.4258 | 2.75 | 0 | P3;P4 | . | P3;P4 |
| 1-68433374-G-A | c.1129-1789C>T | 0.4176 | 5.56 | 0 | P3;P4 |  | P3;P4 |
| 1-68432627-A-AG | c.1129-1043dup | 0.001806 | 0.528 | 0 |  |  | P5 |
| 1-68432081-G-A | c.1129-496C>T | 0.1272 | 0.078 | 0 | P3;P4 |  | P3;P4 |
| 1-68431744-G-A | c.1129-159C>T | 0.0005721 | 3.83 | 0 | P3 |  | P3 |
| 1-68430961-G-A | c.1450+104C>T | 0.3957 | 5.3 | 0 | P3;P4 |  | P3;P4 |
| 1-68429835-G-A | c.1543C>T | 0.00002355 | 26.6 | 0 | P2 | P2 | P2 |
| 1-68429165-C-T | c.*611G>A | 0.4279 | 0.447 | 0 | P3;P4;P5 |  | P3;P4;P5 |
| 1-68429050-G-C | c.*726C>G | 0 | 0.014 | 0 | P3;P4;P5 |  |  |
| 1-68429047-G-GCT | c.*727_*728dup | 0.4276 | 0.078 | 0 | P3;P4;P5 |  | P3;P4;P5 |
| 1-68428861-G-C | c.*915C>G | 0.4284 | 0.454 | 0 | P3;P4;P5 |  | P3;P4;P5 |
